# The Effect of Transcranial Direct Current Stimulation (tDCS) on Cocaine Addiction: A Narrative Review

**DOI:** 10.3390/jcm12206511

**Published:** 2023-10-13

**Authors:** James Chmiel, Justyna Chojdak-Łukasiewicz, Jerzy Leszek

**Affiliations:** 1 Institute of Neurofeedback and tDCS Poland, 70-393 Szczecin, Poland; 2 Department and Clinic of Neurology, Wrocław Medical University, 54-235 Wrocław, Poland; 3 Department and Clinic of Psychiatry, Wrocław Medical University, 54-235 Wrocław, Poland

**Keywords:** cocaine, crack, addiction, transcranial direct current stimulation, dorsolateral prefrontal cortex, tDCS, neuromodulation, cocaine use disorder

## Abstract

Cocaine addiction is a significant problem worldwide. The development of addiction involves a reward system, which consists of certain brain regions like the ventral tegmental area, nucleus accumbens, and prefrontal cortex. Currently, there are no approved medications for treating cocaine dependence, so researchers are actively searching for effective treatments that can impact the brain. One potential treatment under investigation is transcranial direct current stimulation (tDCS), a non-invasive method of stimulating the brain to modulate its activity. In this review, we explore the use of tDCS in treating cocaine addiction. We found nine relevant articles via a literature search, and the results indicate that applying tDCS to the right dorsolateral prefrontal cortex (DLPFC) holds promise for reducing drug cravings in individuals with cocaine addiction. The review also discusses the possible mechanisms by which tDCS works and provides recommendations for future research in this field.

## 1. Introduction

Cocaine is an alkaloid that occurs naturally in the Erythroxylum coca shrub, native to South America [1]. It is considered one of the most potent psychostimulant drugs and has become the second most widely sold drug globally, following marijuana [2]. According to the annual world drug report, approximately 21.5 million people worldwide, or roughly 0.4 percent of the global population aged 15 to 64, are estimated to have used cocaine [3]. Epidemiological data suggest that the prevalence of cocaine use is steadily increasing [4]. Cocaine is highly addictive and can lead to cocaine use disorder (CUD), a significant public health problem associated with severe health, economic, social, and legal consequences [5,6]. In general, addiction, including CUD, is a complex and chronic condition characterized by the compulsive use of a substance or engagement in a behavior. It typically involves physical and psychological dependence, leading individuals to crave the substance or activity and experience withdrawal symptoms when they try to quit. Cocaine falls under the category of “stimulant drugs” as it enhances mood, increases energy, and promotes alertness [7]. The pharmacological effects of cocaine primarily involve blocking the presynaptic transporters responsible for the reuptake of monoamine neurotransmitters such as dopamine (DA) [8], serotonin (5-HT) [9], and norepinephrine (NE) [10] in the central nervous system. In the brain, various types of 5-HT receptors are found, and these receptors play a role in how endogenous 5-HT affects dopamine. Among these receptors, 5-HT2A and 5-HT2C stand out as potential key players in the connection between 5-HT and cocaine abuse [9]. These receptors are also linked to certain characteristics like impulsivity, which contribute to the development of cocaine use disorder and relapse in humans [9]. Cocaine interacts with various neuromodulatory systems, including glutamate, endocannabinoids, and gamma-aminobutyric acid (GABA), contributing to its highly addictive nature [7,11,12]. Cocaine use is associated with numerous acute and chronic complications. Acute effects include intoxication [7], psychological and behavioral changes [7], and psychiatric effects [13]. Furthermore, cocaine usage leads to long-lasting alterations in the vascular system, increasing the risk of conditions such as heart attacks, atherosclerosis, hypertension, and coronary artery disease [14,15,16]. Other complications include seizures [17], anxiety [18], depression [19], an elevated risk of stroke [20], as well as an increased likelihood of contracting sexually transmitted infections [21]. Cocaine usage also results in irreversible damage to the brain, liver, heart, kidneys, and lungs [22].

Cocaine exists in two forms. One is cocaine hydrochloride, commonly referred to as “coke,” “blow,” or “snow,” which is a fine white crystalline powder soluble in water and typically consumed via intranasal, oral, or intravenous routes. The other form is “crack cocaine” or simply “crack,” which is produced by chemically processing cocaine hydrochloride with substances like ammonium or baking soda and is commonly smoked [23]. Individuals who use crack cocaine often experience more family problems, have a higher likelihood of homelessness, and come from broken homes [24]. Various psychosocial factors such as homelessness, poverty, socioeconomic status, unemployment, and legal issues are predictors of mental and physical health complications associated with cocaine use [14].

Prolonged exposure to cocaine leads to various changes in brain function. Like other substances, cocaine affects the brain by altering the transmission of natural neurotransmitters [8,9,10]. Cocaine addiction is linked to the impaired functioning of the prefrontal cortex, heightened sensitivity of the striatum to monetary rewards, and reduced connectivity beyond the brain’s reward circuit [25]. A meta-analysis of functional magnetic resonance imaging (fMRI) studies revealed that individuals addicted to cocaine exhibit excessive activation in several areas: the right inferior temporal gyrus, right insula, right parahippocampal gyrus, and right temporal pole. Conversely, insufficient activation was observed in the right middle frontal gyrus, right inferior parietal gyrus, left inferior parietal gyrus, left middle occipital gyrus, and right middle frontal gyrus [5]. Studies using voxel-based morphometry (VBM) demonstrated decreased gray matter volume in the temporal pole (specifically the superior temporal gyrus), right insula, and right postcentral gyrus among people with cocaine use disorders. However, the gray matter volume of the right inferior parietal gyri was found to be increased. Further multimodal analysis revealed abnormal enlargement of the gray matter volume in the right inferior parietal gyri (excluding the supramarginal and angular regions) [5].

Patients with cocaine use disorder demonstrate cognitive impairments that are not a direct result of cocaine use [26,27]. Previous studies reported that cocaine users may experience some cognitive deficits predating the onset of cocaine use [26]. Research indicates that chronic cocaine use can lead to enduring neuroplastic changes in the prefrontal cortex (PFC), which contribute to the cognitive impairments observed in individuals using cocaine [27]. Studies conducted on rodents exposed to cocaine have revealed alterations in PFC function, including changes in the availability of D1/D2 receptors and synaptic excitability [28]. These changes in the PFC have been associated with deficits in cognitive flexibility, learning, and memory [27,29].

Currently, there are no approved medications specifically for the treatment of CUD [30]. The most promising pharmacological strategy is based on dopamine agonists, such as long-acting amphetamine and modafinil, and GABA agonists/glutamate antagonists, which include topiramate [14]. Psychosocial interventions remain the standard treatment for CUD [31]. Cognitive behavioral therapy (CBT) [32] and motivational interviewing [33] have demonstrated effectiveness. However, despite the advancements in these therapeutic approaches, many patients do not respond adequately to treatment. Treatment for CUD often faces high dropout rates, and achieving long-term abstinence remains challenging for most patients [31]. Previous efforts to develop new medications for the treatment of cocaine abuse have primarily focused on preventing and reducing the acute effects of the drug [31].

Given the extensive neurological changes induced by cocaine and the limited effectiveness of current treatments, there is an urgent need to explore new therapeutic approaches that directly target the brain. One potential avenue is transcranial direct current stimulation (tDCS), a non-invasive technique that has recently garnered attention in psychiatric disorders, including addiction. tDCS involves modulating cortical excitability by delivering a low-intensity direct current via electrodes placed on the scalp [34]. The tDCS device typically consists of a current generator and at least two electrodes (an anode and a cathode). The electrical currents pass through the skull, influencing membrane dynamics in cortical neurons [35]. The effects on neurons arise from induced changes in resting membrane potential, leading to either depolarization (anode) or hyperpolarization (cathode), which can impact neural firing rates and functional connectivity [36]. The effects of tDCS are influenced by various stimulation parameters [37]. Notably, tDCS can induce long-term neuroplastic effects by modulating synaptic plasticity in glutamatergic neurons via N-methyl-D-aspartate (NMDA) receptor-mediated long-term potentiation (LTP) or long-term depression (LTD) mechanisms [38]. Furthermore, tDCS effects are not limited to the stimulated brain region but can also lead to changes in functionally connected areas [39]. Typically, tDCS sessions last between 5 and 30 min, with electric currents ranging from 0.5 to 2 mA [40]. tDCS is generally well-tolerated, with rare adverse effects [41]. The potential therapeutic application of tDCS has been explored in addiction treatment.

The aim of this study is to provide a narrative review of the role of tDCS in treating the symptoms of cocaine addiction and to examine the effects of tDCS on psychiatric symptoms and cognitive outcomes in cocaine-dependent individuals. Several reviews have investigated the effect of tDCS on addiction, also known as substance use disorder (SUD) [42,43,44,45], with promising results. However, all addictions (nicotine, alcohol, cocaine, methamphetamine, marijuana, and others) are included in these studies, which may result in the misinterpretation of the results for only one substance.

## 2. Methods

### 2.1. Data Sources and Search Strategy

For this narrative review, J.C.-Ł. and J.L. performed an independent online search using predefined criteria. The following combined keywords were used: “transcranial direct current stimulation” OR “tDCS” AND “cocaine” OR “crack”. We considered publications on Pubmed/Medline and Research Gate databases (access date March 2023, publication date from January 2014 to December 2021).

### 2.2. Study Selection Criteria

Studies were included when they met inclusion criteria: (1) adult patients; (2) studies applying tDCS on cocaine/crack dependence; (3) presence of sham (placebo) stimulation.

Studies were excluded if they were any of following: (1) multi-substance dependence; (2) patients with severe medical conditions; (3) articles written in languages other than English; (4) duplicated records.

## 3. Results

**Figure 1:**
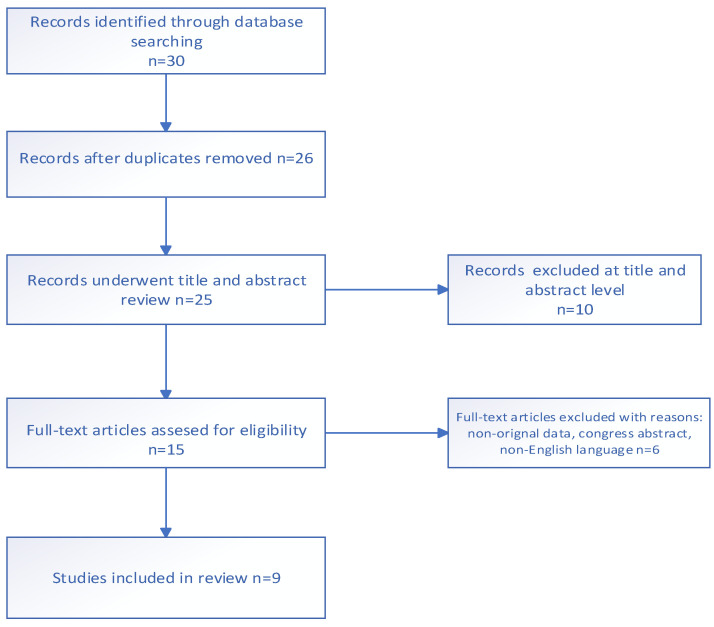
Flow chart of study selection.

The screening process is summarized in a flow chart (Figure 1). During screening, 30 articles were identified. Fourteen studies were excluded by type of publication. Finally, after exclusion, nine articles were included at the end of the selection process. 

A total of 243 patients were enrolled (active tDCS = 128, sham tDCS = 97, control group = 18). Eight studies were randomized controlled trials (RCTs), and one study was open label. Three studies were triple blind, four were double blind, one was single blind, and one was unblinded. Random assignment occurred in eight studies. In the studies [46,47,48], participants were assigned randomly to either of the two groups via a computer-generated block randomization sequence. One study by de Almeida Aramos et al. did not assess the blinding. The applied current ranged from 1.5 to 2 mA. The electrode montage used was bipolar, where both electrodes were placed over the brain, and the same current was delivered via the anode and cathode.

### 3.1. Summary of Included Studies

Table 1 shows the studies included in the analysis. Conti et al. [49] conducted a randomized, controlled clinical study to investigate the effects of tDCS on the dorsolateral prefrontal cortex (DLPFC) in people with cocaine-crack use disorders. Thirteen individuals who were crack cocaine users were randomly assigned to receive either bilateral tDCS (left cathodal/right anodal) or sham tDCS over the DLPFC. The stimulation was administered for five sessions, once a day, every other day. Each tDCS session lasted for 20 min at a current intensity of 2 mA. This study measured brain activity using visual event-related potentials (ERPs) in response to crack-related or neutral cues. The results showed significant differences in the P3-related parameters compared to the group that received active tDCS versus sham tDCS, as well as between single session and repetitive tDCS. After a single session of tDCS, the intensity of the P3 component in the left DLPFC increased during neutral cues but decreased during crack-related cues, which was opposite to the observations in the sham tDCS group. Conversely, repeated sessions of tDCS have been found to increase the distribution of electrical current not only in the DLPFC but also in a wider network of prefrontal areas, including the frontopolar cortex (FPC), orbitofrontal cortex (OFC), and anterior cingulate cortex (ACC), specifically when the subjects were exposed to crack-related cues.

In the randomized, controlled clinical study conducted by Klauss et al. [46], the objective was to investigate whether an increased number of tDCS sessions would further reduce craving and affect relapses to crack-cocaine use. Thirty-five individuals with a crack cocaine addiction were divided into two groups: real tDCS (consisting of 19 participants) and sham tDCS (consisting of 16 participants). The real tDCS group received tDCS with a current intensity of 2 mA for 20 min, with the cathode placed over the left DLPFC and the anode placed over the right DLPFC. Both groups underwent tDCS once a day, every other day, for a total of 10 sessions. Craving levels were assessed using a five-item Obsessive Compulsive Cocaine Scale once a week, including measurements taken before, during (three times), and after the brain stimulation period, spanning approximately 5 weeks. Additionally, relapses to crack cocaine use were monitored for up to 60 days after the participants’ discharge from clinics. The results showed that craving scores decreased progressively over the five measurements in both the sham tDCS and real tDCS groups. The effect sizes, measured using Corrected Hedges’ within-group analysis, were 0.77 for the sham tDCS group and 0.97 for the real tDCS group, indicating a significant reduction in craving. The between-groups effect size was 0.34, favoring the real tDCS group over the sham tDCS group. Regarding relapses to crack-cocaine use after discharge from the hospital, there were similar rates between the two groups within the first 30 days and nearly identical rates after 60 days.

In the randomized, controlled clinical study conducted by Batista et al. [47], the aim was to examine the effects of tDCS on craving in individuals with a crack cocaine addiction. The primary outcome measured was craving, while secondary outcomes included depressive and anxiety symptoms, as well as quality of life. Seventeen male crack cocaine users were randomly assigned to receive five sessions of active tDCS, consisting of a current intensity of 2 mA for 20 min, administered every other day. The results showed that craving scores were significantly reduced in the active tDCS group compared to both the sham tDCS group (*p* = 0.028) and the baseline values (*p* = 0.003) after the treatment. Additionally, the scores related to cravings exhibited a linear decrease throughout the four-week treatment period (prior to treatment, during treatment, and after treatment), specifically in the group that received active tDCS (*p* = 0.047). Changes in anxiety scores differed significantly between the two groups, with the sham tDCS group experiencing an increase and the active tDCS group experiencing a decrease (*p* = 0.03). Additionally, significant differences were observed between the groups in terms of the overall perception of quality of life (*p* = 0.031) and health (*p* = 0.048), with the active tDCS group showing improvements, while the sham tDCS group showed a decline.

The randomized, controlled clinical study conducted by Gaudreault et al. [50] aimed to investigate the effects of tDCS on self-reported craving, anxiety, depression, and quality of life in patients with CUD. Secondary measures encompassed variables such as sleepiness, motivation to modify drug use, emotional state, and cognitive function, specifically impulsivity. Seventeen inpatients diagnosed with CUD were randomly assigned to either the real tDCS condition (with right anodal and left cathodal stimulation) or the sham tDCS condition. The intervention consisted of 15 sessions, with a current intensity of 2 mA administered for 20 min per session. The results showed a trend towards a greater decrease in self-reported craving in the real tDCS group compared to the sham group, although this trend did not reach statistical significance. Both groups exhibited improvements in quality of life and impulsivity over time during treatment. Significant interactions between group and time were observed for daytime sleepiness and readiness to change drug use, indicating that these factors improved only in the real tDCS group. The results of the one-month follow-up indicated temporary effects of tDCS on sleepiness and craving levels.

The randomized, controlled study conducted by Gorini et al. [51] aimed to investigate the effect of tDCS on risky behavior in recently abstinent individuals with cocaine dependence. The study included two groups: 18 dependent cocaine users and 18 control subjects. The researchers examined the effects of left and right stimulation on two risk tasks: the balloon analog risk task (BART) and the game of dice task (GDT). All participants underwent three different stimulation conditions: left anodal/right cathodal stimulation on the DLPFC (LAn+), right anodal/left cathodal stimulation (RAn+), and sham (placebo) stimulation. These stimulation conditions were administered randomly with at least a 48 h interval between each session. The intensity was 1.5 mA, and each session lasted 20 min. The participants performed the BART and GDT before and after each stimulation session. The results showed that activation of the DLPFC (both left and right) led to a reduction in risky behavior during the BART task in both control subjects and cocaine-dependent users. However, the effect of tDCS on the GDT task was more complex. In cocaine users, the right DLPFC anodal stimulation increased safe behavior, while the left DLPFC anodal stimulation increased risk-taking behavior. In control subjects, only the right DLPFC anodal stimulation increased safe bets. 

The randomized, controlled clinical study conducted by Verveer et al. [52] aimed to investigate the effects of tDCS on inhibitory control, risky decision making, craving, and relapse in patients with cocaine use disorder. The participants were assigned to receive ten sessions of either active tDCS or sham tDCS over five consecutive days. The active tDCS group consisted of 29 patients, while the sham group had 30 patients. During the tDCS sessions, the anodal electrode was placed over the F4 region, and the cathodal electrode was placed over the F3 region. In each session, they administered tDCS twice, each time lasting for 13 min at an intensity of 2 mA, with a 20 min break in between the stimulations. Inhibitory control was assessed using a Go-NoGo task, and risky decision making was measured with a two-choice gambling task. These assessments were conducted at baseline, one day after completing all tDCS sessions, and after three months. The researchers also assessed relapse and craving levels during the follow-up period. The findings revealed that active tDCS did not have a significant impact on the frequency of cocaine use and the levels of craving compared to the sham group. Both groups had high relapse rates, with 48.0% in the active tDCS group and 69.2% in the sham group, despite a general decrease in craving during the initial two weeks of treatment. There were no significant effects of tDCS on cognitive functions in the overall analysis. However, in an exploratory analysis focusing on crack cocaine use specifically, the relapse rates were significantly lower in the active tDCS group compared to the sham group.

Conti et al. [53] conducted a randomized, controlled clinical study to investigate the indirect electrophysiological effects of single tDCS on the brains of individuals with cocaine addiction. The study included 13 patients, with 7 receiving active tDCS and 6 receiving sham stimulation. The stimulation was applied either left cathodal/right anodal tDCS or sham stimulation over the DLPFC, with a current intensity of 2 mA and a duration of 20 min. The primary focus of interest in the study was the anterior cingulate cortex (ACC), specifically during the N2 time window (200–350 ms). Event-related potentials were measured in the ACC, while participants were exposed to visual cues related to crack cocaine or neutral cues. The researchers used low-resolution brain electromagnetic tomography (LORETA) to analyze and localize brain activity. The results showed that in the sham group, exposure to crack-related images led to increased ACC activity compared to neutral cues. In contrast, the tDCS group exhibited decreased ACC activity, specifically during the visualization of crack-related cues, but not during the presentation of neutral cues.

De Almeida Ramos et al. [54] conducted a proof-of-concept study to test the effects of tDCS using an opposite montage, with the anode placed on the left DLPFC and the cathode on the right DLPFC on cocaine craving in 11 individuals addicted to crack cocaine. The study involved 10 sessions of tDCS, with a current intensity of 2 mA and a duration of 20 min per session. Cocaine craving was assessed using the Cocaine Craving Questionnaire-Brief (CCQB). This study also assessed cognitive performance using the Montreal Cognitive Assessment (MoCA) and anxiety (Beck Anxiety Inventory). At the baseline and final sessions, participants were exposed to neutral crack images, such as photos of the substance itself and related accessories, to induce craving. The primary outcome measure was the mean reduction in CCQB scores between the baseline and final sessions. The results showed a statistically significant mean reduction of 7.19 (SD 2.18) points in the CCQB scores, indicating a decrease in cocaine craving following the tDCS intervention. However, no significant differences were found in anxiety symptoms and cognitive performance, as assessed using the study measures (*p* = 0.624 and *p* = 0.161, respectively).

Nakamura-Palacios et al. [48] conducted a randomized, controlled clinical study to investigate the effects of tDCS on crack cocaine dependence, focusing on electrophysiological and imaging changes. The study employed a montage with cathodal stimulation on the left DLPFC and anodal stimulation on the right DLPFC, with a current intensity of 2 mA and a duration of 20 min or two sessions of 13 min each for five consecutive days. The researchers analyzed the LORETA data obtained using event-related potentials (ERPs), specifically in the P3 segment (300–500 ms), in response to drug-related cues. Craving levels were assessed using a brief scale adapted from the Obsessive Compulsive Cocaine Use scale. The results demonstrated that repetitive bilateral tDCS led to a decrease in craving among crack cocaine users. Additionally, data obtained from diffusion tensor imaging (DTI) revealed increased DTI parameters in the left connection between the ventromedial prefrontal cortex (vmPFC) and the nucleus accumbens (NAcc) in the tDCS-treated group compared to the sham tDCS group. These DTI parameters, including the number of voxels, fractional anisotropy (FA), and apparent diffusion coefficient (ADC), showed a significant correlation with the reduction in craving following repeated tDCS.

### 3.2. Impact on Cocaine/Crack Craving

Batista et al. [47] utilized a brief scale derived from the Obsessive Compulsive Drinking Scale, consisting of five items (1, 2, 4, 5, and 13), to assess craving in their study. The findings indicated that craving scores exhibited a linear decrease specifically in the tDCS group, starting from the baseline measurement (the week prior to treatment) and continuing to the week following the treatment (linear regression: 4.412 − 0.617X, r^2^ = 0.058, F(1,66) = 4.089).

In the study conducted by de Almeida Ramos et al. [54], crack craving was evaluated using the Cocaine Craving Questionnaire-Brief (CCQB). The results showed a statistically significant mean reduction of 7.19 (standard deviation 2.18) points in the CCQB scores.

In the study of Gaudreault et al. [50], craving was assessed using the Obsessive Compulsive Cocaine Scale and the five-item Cocaine Craving Questionnaire. The results from the analysis of variance (ANOVA) indicated that only the real tDCS group exhibited decreased self-reported craving scores after 15 sessions of stimulation, as measured using the Obsessive Compulsive Cocaine Scale (from 4.4 to 1.9). Similarly, the ANOVA for the five-item Cocaine Craving Questionnaire revealed a decrease in self-reported craving from pre- to post-tDCS (from 12.7 to 6.9). Additionally, the ANOVA for the daily self-report of cocaine craving indicated a linear decrease in craving over the 15 sessions across all subjects.

In the study of Klauss et al. [46], craving was assessed using the Obsessive Compulsive Cocaine Use Scale. The results showed that in the real tDCS group, craving scores were lower at the third, fourth, and fifth time points compared to the first time point and lower at the fourth and fifth time points compared to the second time point. The regression analysis further indicated that craving scores exhibited a linear decrease from baseline (the week before treatment) to the week after treatment in the real tDCS group only. The calculated effect size suggests that when accounting for individual variations, the probability of observing lower craving scores in a crack-cocaine patient after sham tDCS treatment for the final mean score compared to the initial score is 77%, while under real tDCS treatment, it is 84%.

In the study of Nakamura-Palacios et al. [48], craving was assessed using the Obsessive Compulsive Cocaine Use Scale. The results indicated that the active treatment group exhibited a higher decrease in cravings compared to the control group.

In the study by Verveer et al. [52], craving was assessed using an Ecological Momentary Assessment (EMA) scale. The results demonstrated an overall reduction in craving over a period of 14 days following the initial tDCS or sham session, with an average decrease of 1.29. However, there were no significant differences observed in craving over time between the sham and active tDCS groups.

In Conti et al. [49] research, they assessed craving by employing the Brief Cocaine Craving Questionnaire. Their findings indicated that there was no significant alteration in craving following exposure to visual cues associated with crack cocaine use during the ERP procedure.

### 3.3. Impact on Cocaine/Crack Consumption and Relapses

In the study of Klauss et al. [46], relapses in crack cocaine use were observed within 30 days after the completion of 10 sessions of brain stimulation. The frequency of relapses was similar in both the sham tDCS group (41.7%) and the tDCS group (41.2%), with no statistically significant difference between the two groups. Looking at the 60-day follow-up, the frequency of relapses increased in both groups, reaching 66.7% in the sham tDCS group and 52.9% in the real tDCS group, again without significant differences between the groups.

In the study of Verveer et al. [52], the relapse rates within 90 days after the completion of 10 tDCS sessions were found to be high for both the active tDCS group (48.0%, n = 12) and the sham tDCS group (69.2%, n = 18). However, when conducting an exploratory analysis specifically for crack cocaine users, it was observed that the relapse rates were significantly lower in the active tDCS group (41.2%, n = 7) compared to the sham tDCS group (73.7%, n = 14). It is worth noting that this significant difference was not observed among powder cocaine users.

### 3.4. Impact on Quality of Life

In the study of Batista et al. [47], repeated bilateral tDCS targeting the DLPFC was found to decrease craving for crack-cocaine use and improve quality of life, as assessed using the WHOQOL-BREF scale. The scores on the WHOQOL-BREF scale increased after treatment in the real tDCS group (differences of 0.706 and 0.353), while they decreased in the sham tDCS group in the first and second quarters (Q1 and Q2) of the scale (differences of −0.526 and −0.211). Similarly, in the study of Gaudreault et al. [50], both the real tDCS and sham tDCS groups showed an increase in quality of life, as measured using the WHOQOL-BREF scale, from pre-tDCS to post-tDCS sessions (real tDCS: 13 to 14.1, sham tDCS: from 12.6 to 15).

### 3.5. Impact on Depression

In the study of Batista et al. [47], depression symptoms were assessed using the Hamilton Depression Rating Scale (HAM-D). Both the real tDCS and sham tDCS groups showed a decrease in HAM-D scores after treatment (real tDCS: from 5 to 3.2, sham tDCS: from 4.5 to 3.5). However, this decrease was statistically significant (*p* = 0.04) only in the real tDCS group. On the other hand, in the study by Gaudreault et al. [50], tDCS did not have a significant effect on depression symptoms.

### 3.6. Impact on Anxiety

In the study of Batista et al. [47], anxiety symptoms were assessed using the Hamilton Anxiety Rating Scale (HAM-A). The results showed that anxiety scores increased in the sham tDCS group (from 6 to 8.7), approaching statistical significance (*p* = 0.053), while they decreased in the real tDCS group (from 7.6 to 6.4). This contrasting change in anxiety scores between the two groups was statistically significant. On the other hand, both de Almeida Ramos et al. [54] and Gaudreault et al. [50] did not find a significant relationship between tDCS and anxiety in their respective studies.

### 3.7. Impact on Sleepiness

In the study by Gaudreault et al. [50], the researchers observed a decrease in daytime sleepiness among participants who received real tDCS, as measured using the Epworth Sleepiness Scale (from 7.4 to 4.4). Follow-up analyses indicated that individuals in the real tDCS group reported less sleepiness compared to those in the sham tDCS group after completing all 15 tDCS sessions (10.5 vs. 8.9). However, when assessing sleep quality using the Pittsburgh Sleep Quality Index, both groups showed a decrease in overall sleep quality during inpatient treatment, as indicated via a time main effect.

### 3.8. Impact on Drug-Cued Reactivity

In the study conducted by Conti et al. [53], the researchers found that in the sham tDCS group, the ACC activity increased during exposure to crack-related cues compared to the baseline activity before treatment. However, in the tDCS group, the ACC activity decreased during exposure to crack-related cues compared to the baseline activity. No significant changes in ACC activity were observed for neutral images before and after brain stimulation or sham stimulation.

In the study conducted by Conti et al. [49], in the active tDCS group, compared to the baseline activity, they observed a slight increase in brain activity on the left side during the neutral cues condition and no significant change on the right side. During the crack-related cues, they found an increase in brain activity on the right side but a slight decrease on the left side. On the other hand, in the sham tDCS group, compared to their respective baseline values, the activity in the DLPFC during the P3 segment was decreased bilaterally under neutral cues and increased bilaterally under crack-related cues.

In the study conducted by Nakamura-Palacios et al. [48], they found that the ventromedial prefrontal cortex (vmPFC) showed the highest change in drug-related activity during the P3 segment (300–500 ms) as measured using LORETA. Additionally, they conducted fiber tracking analysis using diffusion tensor imaging and observed increases in DTI parameters in the tract connecting the vmPFC and nucleus accumbens (NAcc) following brain stimulation. Importantly, they found a significant correlation between the increase in DTI parameters and the decrease in craving observed after tDCS, suggesting a link between changes in neural connectivity and reduced craving.

### 3.9. Impact on Cognitive Performance

The study conducted by de Almeida Ramos et al. [54] assessed cognitive performance using the Montreal Cognitive Assessment (MoCA). However, they did not find any significant results or effects on cognitive performance based on their analysis.

In the study conducted by Gorini et al. [51], the results showed that stimulation of both the left and right DLPFC resulted in a significant decrease in risk-taking behaviors in both cocaine users and control subjects in the BART task (Sham: from 23.4 to 22.1, RAn+: from 23 to 21, LAn+: from 21.1 to 17.4). In the GDT, right DLPFC activation led to an increase in safe choices for both patients and controls (Sham: from 12.8 to 13, RAn+: from 12.2 to 14). However, left DLPFC stimulation affected the performance of patients, leading to more risk-taking behavior (LAn+: from 14 to 11.2).

In the study conducted by Verveer et al. [52], inhibitory control was assessed using the Go-NoGo task. Both the active tDCS group and the sham group showed improvements in inhibitory control over time, as evidenced by increased accuracy with an average of 5.36, and faster reaction times with an average decrease of 15 ms from pre- to post-intervention. However, when it came to risky decision making, measured using the Two Choice Gambling Task (TCGT), multiple tDCS sessions did not affect participants’ decision making in terms of risk-taking behavior.

### 3.10. Used Montages

In seven studies, anodal stimulation was applied to the right DLPFC. In the study [54], the anodal electrode was positioned on the left DLPFC, and in the study [51], both anodal and cathodal stimulation were applied to the left and right DLPFC. All studies used the 10–20 EEG system to locate brain areas.

### 3.11. Adverse Effects and Safety

Out of the nine studies reviewed, five of them reported mild adverse effects associated with tDCS. These side effects included tingling, headache, heaviness in the head, itching, and scalp burning sensation. In the study conducted by Batista et al. [47], 70.6% of patients from the active tDCS group reported tingling sensations, 17.6% reported a burning sensation, and 5.9% reported a tinnitus sensation after treatment. Conti et al. [49] reported itching sensations at the beginning of sessions, although the exact number of affected individuals was not specified. Gaudreault et al. [50] found that tingling, burning, itching, and fatigue were the most commonly reported side effects in both groups. In the study by Klauss et al. [46], 73.7% of patients in the active tDCS group reported tingling in the scalp, while drowsiness and burning sensation of the scalp were reported by a smaller percentage. As for the study by Verveer et al. [52], adverse effects were mentioned, but no specific information regarding the nature of these effects was provided.

### 3.12. Durability of the tDCS Effects

In the study conducted by Gaudreault et al. [50], a monthly follow-up period was implemented. The results from the five-item OCCS craving subscale indicated lower craving scores in the real tDCS group compared to the sham group (6.4 vs. 2.4). While no significant effects were observed for the five-item Cocaine Craving Questionnaire (sham: 13, tDCS: 12.4), the craving question of the Cocaine Selective Severity Assessment showed a significant time effect. This effect was driven by decreases in cocaine cravings during the stimulation phase, followed by an increase back to baseline levels at the one-month follow-up. Regarding sleepiness, participants reported higher levels of sleepiness at the follow-up assessment compared to the immediate post-tDCS assessment (from 4.4 to 8.9). This suggests that sleepiness values returned to baseline after the one-month follow-up in this group. No significant effects were found for the Pittsburgh Sleep Quality Index, indicating no significant changes in overall sleep quality (from 4.7 to 5). In terms of quality of life, the environment domain of the Quality of Life Questionnaire showed a significant time effect. Values in this domain were higher in both groups after the 15 tDCS sessions and remained elevated after the one-month follow-up (sham: from 15 to 16, tDCS: from 14.1 to 13.9).

In the study of Klauss et al. [46], it was found that craving scores were lower during the one-week follow-up after the intervention. Additionally, during the 60-day follow-up period, the real tDCS group exhibited a greater reduction in relapses compared to the sham tDCS group.

In the study conducted by Verveer et al. [52], the relapse rates within 90 days after the treatment were lower in the active tDCS group (48.0%) compared to the sham tDCS group (69.2%). This suggests that the use of active tDCS may have contributed to a lower likelihood of relapse during the specified follow-up period.

### 3.13. Effectiveness of Blinding

When it comes to the effectiveness of blinding in the study [49], it was not feasible to gather consistent information from the majority of participants. This was primarily due to a significant number of dropouts. The reason for this lack of information was that participants were supposed to be questioned about their experiences when they came back to the outpatient service after completing the tDCS procedures. In the study [46], when patients were asked about their perception of the treatment they had received after completing the treatment, an overwhelming majority of 97% (32 subjects) indicated that they believed they had undergone actual tDCS. In the group that received sham tDCS, all subjects (100%) believed they had received real tDCS, while almost all participants (94.7%) in the real tDCS group also believed they had received the real treatment. A significant portion of the participants, totaling 78.8% (26 in total), which included 78.6% (11 subjects) from the sham tDCS group and 78.9% (15 participants) from the real tDCS group, expressed strong confidence, ranging from very to extremely confident, in their assessment of the treatment they had received. In the study [47], patients were questioned about their beliefs concerning the treatment they had received upon finishing the treatment sessions. A significant majority, or 91.7% (33 subjects), stated that they had been exposed to real tDCS. In contrast, only a small portion, just 8.3% (3 subjects), indicated that they had received sham tDCS. All three of these individuals were from the sham tDCS group. Interestingly, within the sham tDCS group, a notable 84.2% (16 out of 19) claimed to be receiving real tDCS treatment. Conversely, all subjects (100%) from the real tDCS group correctly identified their treatment. Among the 28 subjects, when they were asked about their level of confidence regarding the treatment they had undergone, a significant 82.2% (23 individuals) expressed high levels of confidence, ranging from very to extremely confident. This confidence was distributed between the groups, with 73.3% (11 subjects) from the sham tDCS group and 92.4% (12 subjects) from the tDCS group displaying strong confidence in their assessment of the treatment condition. The remaining studies did not provide information about the degree to which the blinding was successful.

## 4. Discussion

Cocaine addiction is known to have detrimental effects on the brain and can significantly impact behavior and cognition. Consequently, there is a pressing need to develop novel and effective therapeutic approaches to combat this addiction. Transcranial direct current stimulation is one such neuromodulatory intervention that has shown promise in treating addiction syndromes. Several studies have investigated the potential role of tDCS in addressing cocaine addiction. This narrative review aims to explore and summarize these studies, focusing on the effectiveness of tDCS as a treatment for cocaine addiction and examining the effects of tDCS on psychiatric symptoms and cognitive outcomes in cocaine-dependent individuals.

Six studies have examined the impact of tDCS on cocaine/crack craving, and all of them reported positive results in relation to this outcome. These studies consistently demonstrated a decrease in craving levels as assessed using various measurement scales. Furthermore, the observed effects were found to persist for up to one month following treatment. In five studies, cathodal stimulation was applied to the F3 region, while anodal stimulation was administered to the F4 region. It is worth noting that de Almeida Ramos et al. [54] employed a reverse montage, and further research is needed to investigate the effectiveness of this particular stimulation approach.

Two studies investigated the impact of tDCS on relapse rates in individuals with cocaine addiction. In both studies, it was observed that approximately half of the participants experienced relapse during the follow-up period of either 60 or 90 days. However, the relapse rates were found to be lower in the group that received active tDCS compared to those who received sham stimulation. These findings suggest a potential benefit of tDCS in reducing the likelihood of relapse in individuals undergoing treatment for cocaine addiction.

Two studies measured quality of life, and both showed improvements in outcomes. Two studies have investigated the impact of tDCS on depressive symptoms in individuals with cocaine addiction, yielding mixed results. In the study conducted by Batista et al. [47], a decrease in depression measurements was observed following tDCS treatment. However, in the study by Gaudreault et al. [50], no significant effect on depressive symptoms was observed. It is worth noting that the tDCS protocol used in these studies involved a different electrode placement (cathode on F3 and anode on F4) than the typical protocol used for depression treatment. Therefore, further research is needed to determine the specific effects of this electrode placement on depression in the context of cocaine addiction.

Three studies that looked at how tDCS affected anxiety in people who were cocaine dependent produced conflicting findings. The reduction in anxiety was revealed to be a benefit of tDCS in the study by Batista et al. [47]. However, no appreciable effects on anxiety were seen after tDCS treatment in the investigations of de Almeida Ramos et al. [54] and Gaudreault et al. [50]. To fully comprehend the possible effects of tDCS on anxiety in the setting of cocaine addiction, more research is required.

Gaudreault et al. [50] conducted a study to examine the effect of tDCS on sleepiness in individuals with cocaine addiction. The findings revealed that participants who received tDCS reported lower levels of sleepiness compared to the control group. However, it is worth noting that the study also observed a decrease in overall sleep quality among the participants, irrespective of whether they received tDCS or the control treatment. This suggests that while tDCS may alleviate sleepiness, it might not have a positive impact on overall sleep quality.

Three studies have investigated the effects of tDCS on drug-cued reactivity. In the study by Conti et al. [53], it was found that tDCS reduced ACC activity during exposure to crack-related cues. Additionally, during the neutral cues condition, there was a slight increase in activity on the left side of the brain, whereas during crack-related cues, activity increased on the right side but slightly decreased on the left side [49]. In another study by Nakamura-Palacios et al. [48], the highest drug-related activity change in the P3 segment was observed in the ventromedial prefrontal cortex (vmPFC). These findings suggest that tDCS can influence brain responses to drug-related cues. However, further electrophysiological evidence is needed to fully understand the impact of these effects on craving and cocaine consumption.

Three studies have investigated the effects of tDCS on cognitive performance. In the study by de Almeida Ramos et al. [54], no significant impact of tDCS on cognitive performance was detected.

Cocaine addiction is associated with increased risk-taking behaviors and impaired decision making, which are linked to dysfunction in the frontal and striatal brain areas [55,56,57,58]. In a study conducted by Gorini et al. [51], tDCS was found to decrease risk-taking behaviors following both left and right DLPFC stimulation. Additionally, the activation of the right DLPFC in the Game of Dice Task (GDT) led to an increase in safe choices. These findings align with a meta-analysis [59], which suggests that stimulation or inhibition of the left or right DLPFC in healthy individuals results in less risky decision making.

In the study conducted by Verveer et al. [52], the effect of tDCS on inhibitory control was investigated. Inhibitory control is a crucial aspect of SUD and is associated with behavioral adaptability, flexibility, and self-control [60]. Individuals with cocaine addiction have shown impaired inhibitory control, as indicated by studies reporting hypoactivation in brain regions such as the right inferior frontal gyrus, ventrolateral prefrontal cortex, right superior temporal gyrus, and anterior cingulate [61,62,63,64,65,66]. The study in question used a cathodal montage on the left DLPFC and an anodal montage on the right DLPFC, demonstrating improvement in inhibitory control. A meta-analysis by Schroeder et al. [67] suggests that while the right DLPFC stimulation yields an overall null effect, medium effect sizes are observed for right inferior frontal gyrus (rIFG) stimulation. Considering that individuals with cocaine addiction have reduced gray matter volume in the rIFG [68], it is worth exploring the effectiveness of stimulating this region in reducing cravings among people with cocaine use disorders. Furthermore, the effectiveness of rIFG stimulation in reducing cravings in individuals with alcohol addiction has been demonstrated [69].

Our analysis indicates that tDCS stimulation of the DLPFC can effectively reduce cocaine craving. However, the underlying mechanisms of tDCS in the treatment of cocaine addiction remain largely unknown. In the study conducted by Conti et al. [49], the use of left cathodal/right anodal stimulation was shown to reverse the decrease in neuronal activation induced by neutral cues and increase activation induced by crack-related cues in the left DLPFC, as measured by the ERP P3 index. With repetitive tDCS, the inhibitory effect on drug-induced P3 cortical activation shifted towards an increase, particularly in other prefrontal areas such as the FPC, OFC, and ACC, in addition to the DLPFC. The activation of these prefrontal areas may represent a restoration of cortical activity that could be impaired due to repeated crack cocaine use. tDCS may help restore prefrontal cortical processing of environmental stimuli, as suggested by the shift in PFC activation patterns. The right DLPFC is known to play a crucial role in decision making, and the hypoactivation of this brain region is associated with impaired decision-making abilities. The anodal stimulation of the right DLPFC has been shown to reverse this hypoactivation. Furthermore, the bilateral stimulation of the DLPFC, as reported by Nakamura-Palacios et al. [48], can alter the bottom-up processing of evaluating the rewarding properties of drug-related stimuli, potentially influencing behavior toward avoiding drug use. The current body of research examining the effects of tDCS on cocaine addiction is still limited, but there is a growing interest among researchers in exploring this therapeutic approach. Based on the available evidence, tDCS shows moderate effectiveness in reducing cravings for cocaine and crack cocaine, decreasing the likelihood of relapse, and improving quality of life as well as various neuropsychological and cognitive parameters. However, there is a lack of comprehensive electrophysiological data investigating the specific impact of tDCS on brain activity in individuals with cocaine addiction. Advanced neuroimaging techniques, such as fMRI and EEG, have not been extensively employed to elucidate the underlying mechanisms of tDCS in treating addiction. Consequently, definitive conclusions regarding the effectiveness of tDCS as a treatment option for individuals with cocaine dependence cannot be drawn at this time. It is recommended to conduct new, well-designed controlled studies incorporating neuroimaging modalities to confirm the effectiveness of tDCS and gain insights into its neurobiological mechanisms of action.

The current literature suggests that the cathodal stimulation of the left DLPFC and anodal stimulation of the right DLPFC is an effective cortical target for tDCS in the context of cocaine addiction. However, it is worth exploring the effects of the reverse stimulation, specifically the anodal stimulation of the left DLPFC and the cathodal stimulation of the right DLPFC, in future trials. This alternative montage may offer additional insights into the neurobiological mechanisms underlying tDCS and its impact on cocaine addiction. Further investigation is needed to determine the efficacy and potential advantages of reverse stimulation in this population. Additionally, we suggest that future clinical trials consider testing the effectiveness of tDCS for cocaine addiction in at least five sessions per patient. This will ensure that the effects of tDCS will be more visible and measurable.

Cocaine-dependent individuals are known to suffer from cognitive decline. In future trials, it would be valuable to investigate the impact of tDCS on the cognitive functions of individuals with cocaine addiction. Additionally, exploring the potential benefits of combining tDCS with simultaneous cognitive training (CT) is worth considering. By including cognitive training alongside tDCS stimulation, researchers can assess whether the combination of tDCS and CT yields superior outcomes compared to using tDCS or CT alone. This approach may provide insights into the synergistic effects of tDCS and cognitive training in improving cognitive function in individuals struggling with cocaine addiction, as the combination of tDCS with cognitive training is known to have a greater beneficial effect on cognitive function than tDCS alone [70].

The quality of the studies was rated as good, which can be considered a parameter that increases the certainty of the evidence presented. All but one of the included studies were randomized, controlled clinical trials but with small sample sizes. Further randomized, controlled clinical trials with appropriately large active and sham groups are needed to solidify the use of tDCS in cocaine addiction.

This article is a narrative review, and this is a potential limitation of this work. We chose this format because of the small number of highly heterogeneous studies available. Conducting a meta-analysis was not feasible. This leads to a higher potential for bias in the findings. On the other hand, a strength of this study is that it proposes potential mechanisms of action of tDCS in the treatment of cocaine addiction.

## 5. Conclusions

The results of the studies suggest that tDCS shows promise as a potential treatment option for managing cravings in individuals with cocaine addiction. However, the effects of tDCS have been mixed across various outcome measures, and precise conclusions about its overall effectiveness cannot be made at this time. Further high-quality clinical trials incorporating neuroimaging measurements are recommended to enhance our understanding of the underlying mechanisms of tDCS in the context of cocaine addiction. These neuroimaging techniques can provide valuable insights into the specific neural changes associated with tDCS in individuals with cocaine addiction. Additionally, new cortical targets for stimulation have been proposed as potential avenues to enhance treatment outcomes for individuals with cocaine addiction. Exploring these alternative stimulation targets may offer improved functioning and better therapeutic results for individuals struggling with cocaine addiction.

## Figures and Tables

**Table 1:** Studies included in analysis.

References	Participants	Used Test	Double or Single	Brain Region/s Involved	Interventions	Stimulation Site	Current Intensity	Duration	Main Findings in Treatment Group	Follow-Up
Conti et al. (2014) [49]	13 patients 6-real group 7-sham group	BCCQ	double	bilateral tDCS or sham over the DLPFC	five sessions, once a day, every other day	left cathodal/right anodal	2 mA	20 min	significant differences in the P3-related parameters when comparing the group that received active tDCS versus sham tDCS, as well as between single session and repetitive tDCS, there was no significant alteration in craving in BCCQ	
Klauss et al. (2018) [46]	35 patients 19-real group 16-sham group	OCCS	double	bilateral tDCS or sham over the DLPFC	ten sessions, once a day, every other day	left cathodal/right anodal	2 mA	20 min	reduction in cravings in treatment group	after 2 months reduction in relapses in the real tDCS compared to the sham tDCS group
Batista et al. (2015) [47]	36 male patients 17-real group 19-sham group	OCCS	double	bilateral tDCS or sham over the DLPFC	five sessions, once a day, every other day	left cathodal/right anodal	2 mA	20 min	reduction in cravings in treatment group, decreased symptoms of depression, significantly reduction in anxiety	
Gaudreault et al. (2021) [50]	17 patients 8-real group 9-sham group	OCCS, CSSACCQ, HAM-A, HAM-D, WHOQL, ESS, PSQI	double	bilateral tDCS or sham over the DLPFC	fifteen sessions, once a day, every other day	left cathodal/right anodal	2 mA	20 min	the results showed a trend towards a greater decrease in self-reported craving in the real tDCS group compared to the sham group, although this trend did not reach statistical significance. Both groups exhibited improvements in quality of life and impulsivity over time during treatment. Significant interactions between group and time were observed for daytime sleepiness and readiness to change drug use, indicating that these factors improved only in the real tDCS group	the results of the one-month follow-up indicated temporary effects of tDCS on sleepiness and craving levels
Gorini et al. (2014) [51]	36 patients 18-real group 18-control group	BART, GDT	double	bilateral and sham over the DLPFC	3 sessions with 48 h intervals	left anodal/right cathodal and left cathodal/right anodal	1.5 mA	20 min	right DLPFC anodal stimulation increased safe behavior, while left DLPFC anodal stimulation increased risk-taking behavior	
Verveer et al. (2020) [52]	59 patients 29-real group 30-sham group	EMA, Go-NoGo task, TCGT	double	bilateral tDCS or sham over the DLPFC	ten sessions over five consecutive days. twice daily, with a 20 min break between tDCS	left cathodal/right anodal	2 mA	26 min (in double applications of 13 min with 20 min interval in between)	an overall reduction in cravings over a period of 14 days following the initial tDCS or sham session, the relapse rates were significantly lower in the active tDCS group (not observed among powder-cocaine users). Both the active tDCS group and the sham group showed improvements in inhibitory control over time. tDCS sessions did not have an effect on participants’ decision-making in terms of risk-taking behavior	after 90 days risk of relapses was lower in the active tDCS group (48.0%) compared to the sham tDCS group (69.2%)
Conti et al. (2014) [53]	13 patients 7-real group 6-sham group	-	double	bilateral tDCS or sham over the DLPFC	one session	left cathodal/right anodal	2 mA	20 min	significant decrease in the ACC activity (anterior cingulate cortex) after bilateral tDCS	
de Almeida Ramos et al. (2016) [54]	11 patients 11-real group	CCQB, BAI, MoCA	single	bilateral tDCS or sham over the DLPFC	ten sessions	left anodal/right cathodal	2 mA	20 min	statistically significant mean reduction in craving	
Nakamura-Palacios et al. (2016) [48]	23 patients 13-real group 10-sham group	OCCS	double	bilateral tDCS or sham over the DLPFC	five sessions for five consecutive days or two sessions a day	left cathodal/right anodal	2 mA	20 min or in double applications of 13 min with 20 min interval in between	reduction in cravings in treatment group	

DLPCF—Dorsolateral Prefrontal Cortex; BCCQ—Brief Cocaine Craving Questionnaire; OCCS—Obsessive Compulsive Cocaine Use Scale; CCQB—Cocaine Craving Questionnaire-Brief; CSSA—Cocaine Selective Severity Assessment; SRC—Self-reported Cocaine Use Test; BART—Balloon Analog Risk Task; GDT—Game of Dice Task; TCGT—Two Choice Gambling Task, BAI—Beck Anxiety Inventory; MoCA—Montreal Cognitive Assessment; CCQ—Cocaine Craving Questionnaire; HAM-A—Hamilton Anxiety Rating Scale; HAM-D—Hamilton Depression Rating Scale; WHOQL—World Health Organization Quality of Life; ESS—Epworth Sleepiness Scale; PSQI—Pittsburgh Sleep Quality Index; EMA—Ecological Momentary Assessment.

## Data Availability

No new data were created or analyzed in this study. Data sharing is not applicable to this article.
